# Genome- and transcriptome-derived microsatellite loci in lumpfish *Cyclopterus lumpus*: molecular tools for aquaculture, conservation and fisheries management

**DOI:** 10.1038/s41598-019-57071-w

**Published:** 2020-01-17

**Authors:** Simo N. Maduna, Adam Vivian-Smith, Ólöf Dóra Bartels Jónsdóttir, Albert K. D. Imsland, Cornelya F. C. Klütsch, Tommi Nyman, Hans Geir Eiken, Snorre B. Hagen

**Affiliations:** 1 Norwegian Institute of Bioeconomy Research (NIBIO), Division of Environment and Natural Resources, P.O. Box 115, NO-1431 Ås, Norway; 2 Norwegian Institute of Bioeconomy Research (NIBIO), Division of Forestry and Forest Resources, P.O. Box 115, NO-1431 Ås, Norway; 3 Akvaplan-niva, Iceland Office, Akralind 4, 201 Kópavogur, Iceland; 4 Department of Biosciences, University of Bergen, 5020 Bergen, Norway

**Keywords:** Genetic markers, Conservation genomics

## Abstract

The lumpfish *Cyclopterus lumpus* is commercially exploited in numerous areas of its range in the North Atlantic Ocean, and is important in salmonid aquaculture as a biological agent for controlling sea lice. Despite the economic importance, few genetic resources for downstream applications, such as linkage mapping, parentage analysis, marker-assisted selection (MAS), quantitative trait loci (QTL) analysis, and assessing adaptive genetic diversity are currently available for the species. Here, we identify both genome- and transcriptome-derived microsatellites loci from *C. lumpus* to facilitate such applications. Across 2,346 genomic contigs, we detected a total of 3,067 microsatellite loci, of which 723 were the most suitable ones for primer design. From 116,555 transcriptomic unigenes, we identified a total of 231,556 microsatellite loci, which may indicate a high coverage of the available STRs. Out of these, primer pairs could only be designed for 6,203 loci. Dinucleotide repeats accounted for 89 percent and 52 percent of the genome- and transcriptome-derived microsatellites, respectively. The genetic composition of the dominant repeat motif types showed differences from other investigated fish species. In the genome-derived microsatellites AC/GT (67.8 percent), followed by AG/CT (15.1 percent) and AT/AT (5.6 percent) were the major motifs. Transcriptome-derived microsatellites showed also most dominantly the AC/GT repeat motif (33 percent), followed by A/T (26.6 percent) and AG/CT (11 percent). Functional annotation of microsatellite-containing transcriptomic sequences showed that the majority of the expressed sequence tags encode proteins involved in cellular and metabolic processes, binding activity and catalytic reactions. Importantly, STRs linked to genes involved in immune system process, growth, locomotion and reproduction were discovered in the present study. The extensive genomic marker information reported here will facilitate molecular ecology studies, conservation initiatives and will benefit many aspects of the breeding programmes of *C. lumpus*.

## Introduction

Lumpfish, or lumpsucker, *Cyclopterus lumpus* Linnaeus, 1758 (Cottoidei: Cyclopteridae) is a semi-pelagic teleost species commonly found across the North-Atlantic Ocean^1^ and to a lesser extent in the Mediterranean Sea^2^. Females of this species are commercially exploited for their ripe egg masses (roe) which are sold as caviar in the European Union and Asian markets. However, both sexes are often incidentally captured as bycatch in other major fisheries^3,4^. *Cyclopterus lumpus* also plays an increasingly indispensable role in salmonid aquaculture. As a ‘cleaner fish’ it forms an important biological control measure for sea lice (*Lepeophtheirus salmonis* Krøyer, 1838) on Atlantic salmon (*Salmo salar* Linnaeus, 1758) aquaculture farms in the Northern Hemisphere^5–8^. The importance of *C. lumpus* in both fisheries and aquaculture has therefore motivated genetic studies to delineate the population structure and define management units in the wild^9–12^. The available panel of 22 microsatellite markers for *C. lumpus* can efficiently address many questions in molecular ecology^13^; however, the current panel is not sufficient for downstream applications such as linkage mapping, parentage analysis, identification of quantitative trait loci (QTL), marker-assisted selection (MAS) and for studying adaptive genetic diversity.

Microsatellites, also known as Short Tandem Repeats (STRs), are repeated motifs of one to six nucleotides that have a characteristic mutational behaviour resulting in repeat number differences within and amongst individuals^14–16^. The multi-allelic nature of STRs is a consequence of their elevated mutation rates as compared to other marker types^16–18^. However, STR mutation rates are highly variable among organisms, loci, repeat types, and even alleles at a locus^19–23^. STRs are co-dominantly inherited (each allele can be scored), ubiquitously distributed in eukaryotic genomes, and occur in both coding and non-coding regions^14,17,24^. Accordingly, STRs are classified into two types based on their location in the genome: Type I STR loci are located within functional genes, while Type II STR loci are located within non-coding intergenic regions^14,24^. Type I STRs are commonly isolated from transcribed regions or expressed sequence tags (EST) obtained through transcriptome sequencing (EST-STRs hereafter), while Type II STRs are derived from non-transcribed genomic regions through genome sequencing (genomic STRs, g-STRs hereafter)^25–28^. Genomic-based STRs are still among the most frequently used genetic markers for inferring spatial patterns of population structure, genetic diversity, migration rates, effective population size and kinship within species since, in most cases, these markers are selectively neutral^17,18^. By contrast, EST-based STRs are gene-linked markers (*i.e*., they reside within or proximal to functional genes) that may be subject to selection. Accordingly, EST-STRs have a higher probability of association to phenotypic effects, or to causal mutations, therefore, they are also useful in studying adaptive processes within and between species^25–27^. In addition, transferability of STRs amongst congeneric and confamilial (target) species has been reported in many taxonomic groups, with the rate of success often correlating with evolutionary distance between the source and target species^29–32^. Therefore, STRs remain one of the most informative and versatile markers available for genetic investigations into ecosystem-, population- and individual-level questions^17,27,28,33^.

Recent advances in high throughput sequencing (HTS) techniques have led to innovative labour- and cost-effective methods for discovering and genotyping STRs in species for which little or no sequence information is available^34–36^. Similar to the approaches used for the simultaneous detection and genotyping (genotyping by sequencing, GBS) of single nucleotide polymorphisms (SNPs^37^), STRs can now be genotyped faster and cheaper using HTS-based microsatellite-GBS approaches instead of traditional capillary electrophoresis^36^. Microsatellite-GBS approaches have rapidly advanced and been applied in population-genetic studies of Atlantic cod *Gadus morhua* Linnaeus, 1758^38^, boarfish *Capros aper* Linnaeus, 1758^39^, muskrat *Ondatra zibethicus* Linnaeus, 1766^40^, fruit fly *Drosophila melanogaster* Meigen, 1830^35^, red deer *Cervus elaphus* Linnaeus, 1758^35^, brown bear *Ursus arctos* Linnaeus, 1758^41^ and chimpanzee *Pan troglodytes* Blumenbach, 1776^36^. Nonetheless, these methods require prior knowledge on STR loci and their variation and, most critically, information on the flanking sequences for primer design^36,38,39^. Background information for designing primers can be obtained by using reduced-representation sequencing approaches such as double-digest (dd) restriction site associated DNA sequencing (RADseq), which offers a relatively fast and cheap option for recovering large amounts of sequence data^42,43^. Recently, ddRADseq has proved useful in discovery of STRs in numerous non-model species (e.g.^44–46^).

In this work, our aims were twofold: (i) to investigate the distribution and nucleotide composition of microsatellite sequences in *C. lumpus*; and (ii) to expand the STR marker base for *C. lumpus* by developing a larger set of genomic-based and EST-derived STRs using an in-silico approach. The genomic information generated for *C. lumpus* will facilitate linkage- and QTL mapping as well as marker-assisted selection for important traits, particularly to those relating to the genetic patterns of both adaptive and neutral variation in wild populations, but also for gaining invaluable insight into the impact of escapees from aquaculture farming.

## Results

Reduced-representation ddRAD sequencing of two individuals of *Cyclopterus lumpus* in a two sequencing runs on the Ion PGM™ NGS platform resulted in a total of 990,653 quality filtered single-end reads (25–532 bp sequence length; 46 percent GC content). The transcriptome assembly based on 13*C. lumpus* individuals^47^, comprised of 346,430 transcripts from 221,659 trinity genes, while the *de novo* transcriptome assembly of the Fish-T1K data consists of 49 million assembled bases in 98,767 transcripts from 89,342 trinity ‘genes’. The median transcript length was 362 bases, average length 550 bases and N_50_ 669 bases. Assembly of unigenes by CAP3 from the transcriptome assembly of *C. lumpus* by Eggestøl *et al*.^47^ generated a total of 255,957 unigenes (52,671 contigs and 203,286 singletons), while for the Fish-T1K transcriptome a total of 53,703 unigenes (23,831 contigs and 29,872 singletons) were produced.

**Figure 1:**
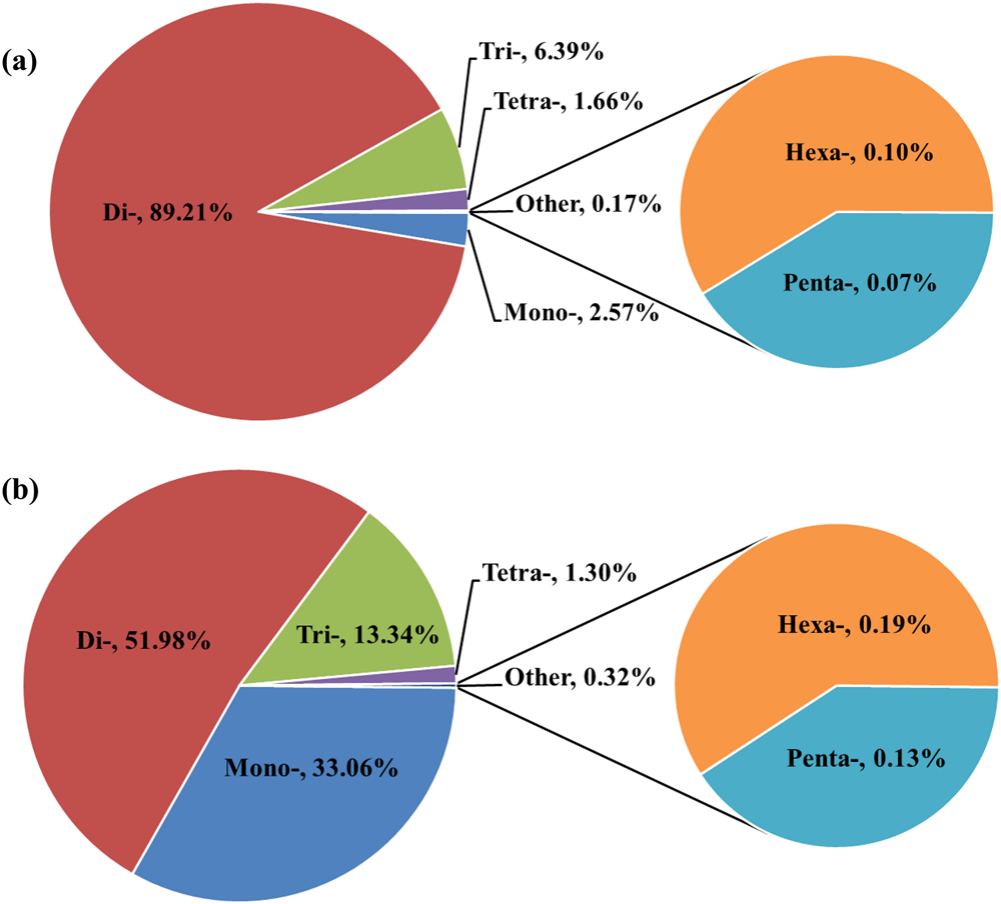
Relative frequencies of different motif length classes in (**a**) g-STRs and (**b**) EST-STRs of *Cyclopterus lumpus*.

**Figure 2:**
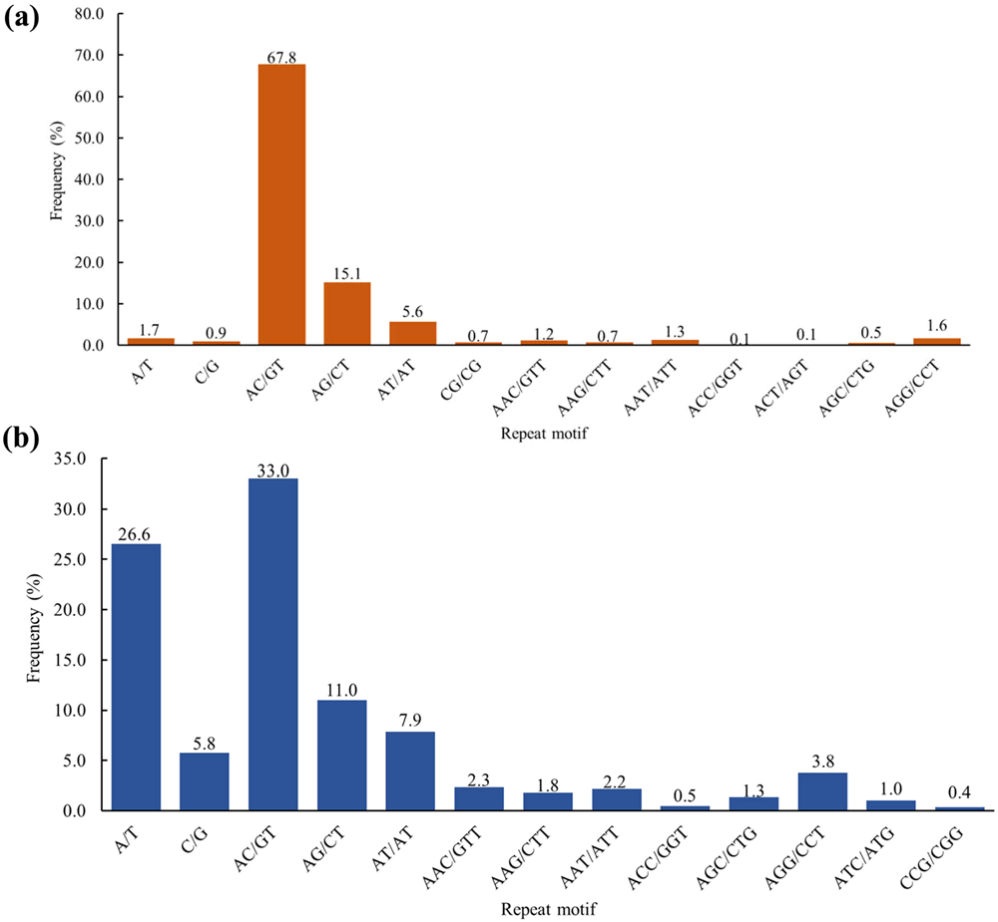
Relative frequencies of the 13 most abundant non-redundant repeat motifs in (**a**) g-STRs and (**b**) EST-STRs in *Cyclopterus lumpus*.

We obtained 2,346 STR-containing consensus sequences by analysing the ddRADseq data generated from two unrelated individuals of *Cyclopterus lumpus*. A total of 1,791 sequences contained STRs of different motif types, and 555 sequences contained two STRs. STR detection by the QDD-VM pipeline revealed a total of 3,067 g-STRs, of which 2,387 (77.83 percent) were simple repeat motifs and 680 (22.17 percent) were in compound formation (Table 1). Dinucleotide repeat motifs were most frequent (2,736; 89.21 percent), followed by trinucleotide (196, 6.39 percent), mononucleotide (79; 2.57 percent), and tetranucleotide (51; 1.66 percent) repeats, while only three (0.1 percent) hexanucleotide and two (0.07 percent) pentanucleotide repeat units were found (Fig. 1a). The distribution of g-STRs to different repeat motif length classes estimated by MISA varied from 5 to 42, with five repeats (25.69 percent) being most common, followed by 15+ (14.31 percent), six (12.16 percent) and seven (7.76 percent) repeats (Table 2). The frequency of the dinucleotide repeat motifs was highly represented across repeat length classes. A total of 33 types of consensus (non-redundant) repeat motif were found among the STR-containing sequences (Table S1, Supplementary Material). The dominant repeat motif type was dinucleotide (AC/GT)_*n*_, where n refers to the number of times the unit is repeated, with a frequency of 67.80 percent (2,027), followed by (AG/CT)_*n*_ (463, 15.10 percent), and (AT/AT)_*n*_ (173, 5.60 percent). Trinucleotide repeats were only represented by an overall frequency of 6.10 percent among the 13 most abundant repeat motif types (Fig. 2a). Of the 2,346 STR-containing sequences, 723 were suitable for microsatellite primer design, and a total of 8,313 primers targeted at producing different amplicon sizes (multiple primer pairs) per locus were successfully designed ((Table 1); Table S2, Supplementary Material).

**Table 1:** Summary of the *in silico* search for STRs in the *Cyclopterus lumpus* genome and transcriptome, respectively. Figure in parenthesis show the total number of STR-containing post-annotation filtering.

Search parameters	Genomic	Transcriptomic
Total number of sequences examined	990,653	322,381
Total length of examined sequences (bp)	213,385,867	418,639,584
Total number of unique reads (reduced by QDD)	2,346	255,957
Total number of identified STRs	3,067	231,556
Number of STR-containing sequences	2,346	116,555
Number of sequences containing more than 1 STR	555	57,717
Number of STRs present in compound formation	680	38,550
Number of STR-containing sequences with primers	723 (394)	6,203

**Table 2:** Distribution of *Cyclopterus lumpus* g-STRs to different repeat motif length classes.

Repeats motif	Number of repeats											
	5	6	7	8	9	10	11	12	13	14	15	15+
Mononucleotide						30	15	8	11	6	4	5
Dinucleotide	657	333	206	207	164	175	137	153	108	98	67	431
Trinucleotide	101	31	25	20	7	2	2	5	2	1	0	0
Tetranucleotide	26	9	6	1	1	1	2	1	0	0	1	3
Pentanucleotide	1	0	1	0	0	0	0	0	0	0	0	0
Hexanucleotide	3	0	0	0	0	0	0	0	0	0	0	0
**Total**	**788**	**373**	**238**	**228**	**172**	**208**	**156**	**167**	**121**	**105**	**72**	**439**
**Type percentage**	**25.69**	**12.16**	**7.76**	**7.43**	**5.61**	**6.78**	**5.09**	**5.45**	**3.95**	**3.42**	**2.35**	**14.31**

Using the QDD-VM pipeline to also screen the transcriptome of *C. lumpus* for Type I STRs, we isolated a total of 116,555 sequences containing STRs of different motif types, where 57,717 sequences contained between two and 15 STRs, as expected for a transcriptome assembly. QDD-VM detected a total of 231,556 EST-STRs, of which 193,006 (83.35 percent) represented simple repeat motifs and 38,550 (16.65 percent) were in compound formation (Table 1). The relative abundance of STRs was estimated to be 630.43 loci/Mb. Dinucleotide repeat motifs were most frequent (120,353; 51.98 percent), followed by mononucleotide (76,565; 33.06 percent), trinucleotide (30,900; 13.34 percent), and tetranucleotide motifs (3,005; 1.30 percent), while only 433 (0.19 percent) hexanucleotide and 300 (0.13 percent) pentanucleotide repeat units were found (Fig. 1b). The distribution of EST-STRs to different repeat motif length classes ranged from 5 to 83, with a maximum frequency for five repeats (23.86 percent), followed by 10 (14.35 percent), six (12.05 percent) and 15+ (11.7 percent) repeats (Table 3). Mononucleotide repeat motifs were highly represented across repeat length classes, followed by dinucleotides and trinucleotides. We found a total of 145 types of non-redundant repeat motif among the STR-containing sequences (Table S3, Supplementary Material). The dominant repeat motif type was dinucleotide (AC/GT)_*n*_ (75,479; 33.00 percent), followed by (A/T)_*n*_ (62,984; 26.60 percent), and (AG/CT)_*n*_ (25,631; 11.00 percent). Trinucleotide repeats were only represented by an overall frequency of 13.30 percent among the 13 most abundant repeat motif types (Fig. 2b). From the 116,555 STR-containing sequences, primers could be designed for 6,203 sequences, resulting in a total of 117,374 primers ((Table 1); Table S4, Supplementary Material).

**Table 3:** Distribution of *Cyclopterus lumpus* EST-STRs to different repeat motif length classes.

Repeats motif	Number of repeats											
	5	6	7	8	9	10	11	12	13	14	15	15+
Mononucleotide						26,626	15,798	9,731	6,569	4,150	3,321	10,370
Dinucleotide	39,548	19,578	12,715	10,011	7,856	5,628	5,661	1,625	80	217	1,250	16,184
Trinucleotide	13,690	7,555	4,583	1,337	98	858	760	600	469	268	214	468
Tetranucleotide	1,695	633	51	136	124	89	58	45	19	66	24	65
Pentanucleotide	176	31	32	25	6	7	7	6	4	2	1	3
Hexanucleotide	136	97	64	51	27	19	12	8	9	5	0	5
**Total**	**55,245**	**27,894**	**17,445**	**11,560**	**8,111**	**33,227**	**22,296**	**12,015**	**7,150**	**4,708**	**4,810**	**27,095**
**Type percentage**	**23.86**	**12.05**	**7.53**	**4.99**	**3.5**	**14.35**	**9.63**	**5.19**	**3.09**	**2.03**	**2.08**	**11.7**

Local BLAST search of the 22 microsatellites reported by Skirnisdottir *et al*.^13^ against our ddRADseq dataset returned significant hits only for loci *Clu11* (JX485370.1) and *Clu40* (JX485383.1), with a 100 percent and 99.2 percent sequence similarity, respectively (Table S5, Supplementary Material). However, the STR contigs containing *Clu11* and *Clu40* did not meet our criteria for primer design in our dataset. A similar search for previously-reported *C. lumpus* loci in our EST dataset yielded significant hits for six loci: *Clu07*, *Clu11*, *Clu19*, *Clu36*, *Clu40*, and *Clu45* (Table S5). As for the two previously-described g-STRs, these loci were not part of the sequences used for primer design in our study.

**Figure 3:**
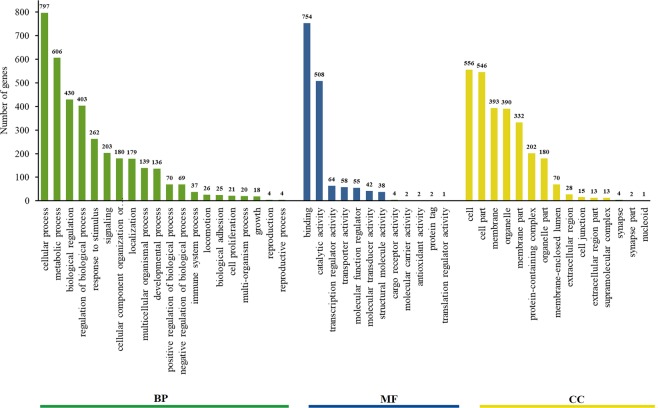
Annotation of STR-containing EST unigenes in *Cyclopterus lumpus* according to their biological function, namely biological process (PB), cellular component (CC), and molecular function (MF) and respective subcategories.

Functional annotation of EST-STR-containing unigenes based on the BLASTx analysis through BLAST2GO resulted in a total of 4,931 annotations. Through mapping, we could extract GO terms for assigning gene products into three categories, biological process (BP), cellular component (CC), and molecular function (MF). According to the GO analysis, 2,009 unigenes were assigned to the BP category, 1,789 to the MF category and 1,119 to the CC category. The small discrepancy between total annotation and the number of unigenes is expected for transcriptome assemblies, since contigs may contain multiple gene regions that are assigned to different categories. Within the BP category, genes involved in cellular, metabolic and biological regulation comprised the largest portion (Fig. 3), while in the CC category the greatest number of genes were found to encode cellular components and cell parts (Fig. 3). Likewise, many sequences in the MF category encode proteins with binding and enzymatic activity (Fig. 3). We recovered a total of 135 annotations for g-STR-containing sequences, and a local BLAST search of g-STRs in our EST-STR data base yielded 332 significant hits. After removing these sequences, we could retain a final set of 394 actual g-STRs.

## Discussion

Developments in high-throughput sequencing (HTS) technologies have afforded us with the opportunity to obtain genomic and transcriptomic sequences suitable for isolating vastly larger sets of STRs distributed across genomes compared to previous enrichment methods involving oligonucleotide hybridisation and cloning^48^. We implemented a STR discovery pipeline suitable for detecting STRs from either assembled (contigs or scaffolds) or non-assembled sequences. From assembled sequences STRs are extracted along with their flanking regions for primer design. Non-assembled sequences are first filtered to remove adaptors and eliminate short reads (<80 bp in our case) prior to identifying reads with STRs. Importantly, STRs can be detected at any given sequence length (a user-defined parameter). Although, to produce functional markers *i.e*. those loci that will successfully amplify *in vitro*, the optimum read length range is 150–500 bp for non-assembled sequences^49^. In the present study, 30 percent of the STR-containing non-assembled ddRAD sequences were suitable for primer design while 5 percent of the STR-containing transcriptome assembly sequences were suitable for primer design, indicating that the length and sequence properties of the STR-flanking region is also an important parameter. In all, we report on the identification of a total of some 232,000 genome-wide STR loci in *C. lumpus* based on an *in silico* STR development approach. As expected, *ca*. 98 percent of the genome-wide STRs were isolated from the transcriptome assembly compared to the data generated from reduced-genome sequencing. Nevertheless, the combinatorial use of genomic and transcriptomic sequences allowed us to isolate and design primers for STRs located in both coding and non-coding regions of the *C. lumpus* genome.

In *C. lumpus* the most common STR lengths were five, six and 10. We note that dinucleotide repeats motifs were dominant in the genome-derived STRs accounting for 89 percent, which is similar to previous studies on other fish^50,51^. Dinucleotide repeats were overrepresented also in EST-derived STRs, where they accounted for 52 percent, indicating that dinucleotide repeats are the dominant motif in the genome of *C. lumpus*. This trend is in accordance with EST-STR distributions described earlier in several fish species, including the channel catfish *Ictalurus punctatus* Rafinesque, 1818 (72 percent), killifish *Fundulus heteroclitus* Linnaeus, 1766 (52 percent), Japanese medaka *Oryzias latipes* Temminck and Schlegel, 1846 (47 percent), platyfish *Xiphophorus maculatus* Günther, 1866 (78 percent), zebrafish *Danio rerio* Hamilton, 1822 (64 percent)^52,53^ and crucian carp *Carassius auratus* (as defined by Zheng *et al*.^54^ Linnaeus, 1758 (44 percent)^54^. Dinucleotide repeat motifs in *C. lumpus* were predominantly composed of AC/GT, which has been found to be the case also in all the aforementioned fishes except the killifish, in which the most common motif was AT/TA. The proportion of trinucleotide motifs in *C. lumpus* likewise resembles findings from other fishes, however, the nucleotide composition appears to differ since the most abundant trinucleotide motif in *C. lumpus* is AGG/CCT, while in catfish the two most abundant types are ATA and TTA, and in zebrafish, killifish and crucian carp the AAT/TTA motif dominates^52,53^. These differences suggest that the predominant repeat motif in fish is by no means consistent across species and taxa.

Functional annotation of the STR-containing EST unigenes revealed that the majority of these encoded for proteins involved in protein-binding and catalytic reactions. This is consistent with earlier studies providing compelling evidence that STRs, especially the AC repeat motif, play an important role in protein-binding and transcriptional activity^55–57^. The recently assembled transcriptome of *C. lumpus* by Eggestøl and co-workers^47^ was targeted at identifying and mapping the components of the immune system involved in early immune responses of leukocytes following *in vitro* exposure to the pathogenic bacterium *Vibrio anguillarum* O1. As such, we anticipated to also uncover STRs linked to genes involved in immunity. Interestingly, an investigation into the cleaning behaviour (sea lice grazing efficacy) and disease resistance in several families of *C. lumpus* showed significant difference among families^6^. Inquiries into the genetic basis of grazing efficacy and disease resistance traits have been hampered by the limited available genomic resources for *C. lumpus*. In the present study, we also discovered STRs linked to genes involved in immune system process, growth, locomotion and reproduction to aid in such endeavours. We also noticed that genome-derived STRs to some degree overlapped with transcriptome-derived STRs, both in our g-STR dataset and in that of Skirnisdottir *et al*.^13^, indicating that genome-derived STRs can also include Type 1 STR loci. To this end, our bioinformatic framework allows for distinguishing Type I and -II STR loci in genome-derived STR databases and could be useful for developing both STR types in other species.

Khimoun *et al*.^27^ assessed whether the patterns and levels of genetic diversity within and between bird populations are similar for EST- and g-STRs, and investigated how the levels of differentiation influence the relative efficiency of the respective marker types. They found that when there is strong genetic differentiation, inferred population-genetic structures were similar for both marker types, but that g-STRs slightly outperformed EST-STRs when differentiation was moderate. On the contrary, the study then provides compelling evidence that EST-STRs have a higher resolution in detecting weak population genetic structure compared to g-STRs. This pattern is consistent with earlier studies in plants^26,58^ and, more recently, in sharks^28^. The previous studies also show that when using EST- and g-STRs as a single panel, this can result in the underestimation of the degree of population structure, especially when genetic structuring is weak^27,28^. Functional annotation of STR-containing sequences during marker development is therefore crucial, so that the actual distribution of anonymous g-STRs and EST-STRs can be accurately quantified and then properly applied in downstream genetic analyses.

Bioinformatics workflows for extracting STR locus-specific sequences from HTS dataset are gradually becoming available to facilitate genotyping-by-sequencing (GBS) of STRs^36,39,41^. Barbian and co-workers^36^ compared the performance of capillary electrophoresis and HTS to validate and improve the STR-GBS approach. In that study, it was shown that the GBS approach identified new alleles based on sequence differences that were previously masked by size homoplasy. The large STR primer base reported in the present study for *C. lumpus* used the revised primer design parameters of Meglécz *et al*.^49^ which were empirically validated to improve genotypic success rates. Although the present study did not involve the *in vitro* validation of the primers to determine amplification efficiency and the level of polymorphism (*i.e*., number of alleles) at each of the loci, Meglécz *et al*.^49^ reported that the target region complexity had no effect on the polymorphism of STRs, and that the levels of polymorphism increased from di- to tetra-nucleotide repeat motifs in their two focal species. Furthermore, as HTS data generated from a larger panel of individuals becomes available for *C. lumpus*, it will be possible to possible to perform electronic PCR (e-PCR^59^) and extract locus-specific genotypes without the need to perform *in vitro* experiments. For instance, the specificity of e-PCR (*in silico*) amplification (compared to BLAST) previously enabled breeders to identify the map positions of STRs in rice *Oryza sativa* L^60^. and potato *Solanum tuberosum* L.^61^, and anchor the STR loci within linkage groups without the need to perform additional PCR reactions. Moreover, e-PCR was used successfully to align the STR loci on the linkage map of *Brassica napus* L. to the genome of *B. rapa* L. and *B. oleracea* L. to identify candidate genes of QTLs for seed weight through comparative mapping of these *Brassica* species to *Arabidopsis thaliana* L.^62^. Therefore, STRs provide significant utility in that they provide a source of genetic variation that has a higher mutation rate and transferability success rate across populations and species than for SNPs^31,63^.

The *C. lumpus* STR database reported in the present study provides valuable molecular markers to the scientific community for a myriad of downstream applications, such as linkage mapping, parentage analysis, marker-assisted selection (MAS), quantitative trait loci (QTL) analysis, and assessing adaptive genetic diversity in this commercially valuable fish. Moreover, the large STR primer sets reported here readily allows for exploring the cost-efficient HTS-based STR genotyping-by-sequencing approach in *C. lumpus* or even data generated from HTS-based SNP genotyping experiments.

## Methods

### Sampling and DNA extraction

We obtained finclip samples of two *C. lumpus* individuals, of which one originated from southern Norway (Mandal, N 57.99 E 7.48) and one from northern Norway (Hekkingen, N 69.37 E 17.48). The finclip samples used in our study were from dead specimens caught during normal fishing activity and, therefore, no approval from the local ethics committee was necessary. We stored the samples at 4 degrees Celsius in absolute ethanol. To extract total genomic DNA from the samples, we used the DNeasy Blood and Tissue Kit following the manufacturer’s instructions (Qiagen).

### ddRAD library preparation and NGS data processing

DNA extracts were quantified with the Qubit Broad Range dsDNA Assay (Thermo Fisher Scientific), and then diluted to standardised working concentrations in nuclease-free water. Library preparation was then performed following the modified ddRAD digestion-ligation protocol of Vivian-Smith and Sønstebø^43^. We then performed a combined double-digest and ligation reaction for each individual using 200 ng of DNA. This reaction was composed of the high fidelity restriction endonucleases PstI-HF and NdeI-HF (specific for CTGCA|G and CA|TATG restriction motifs, respectively; New England Biolabs), together with ligation reaction using the modified adapters P1 and A in a total volume of 60 micro-liters, using the NEB 4 buffer as previously described^43^. Next, we pooled the barcoded libraries and purified them with Agencourt AMPure XP Beads (Cat. No. A63881; Beckman Coulter). The pooled library was then resolved on a pre-cast 1.5 percent agarose gel in a Pippin Prep automated electrophoretic system (Sage Science) set to recover 400–600 bp fragments. Subsequently, we size selected for two fragment-length ranges and constructed two libraries of 443–493 bp and 493–553 bp, corresponding to insert sizes of 340–390 and 390–450 bp, respectively. To check the quality and quantity of each library size selection, we used the BioAnalyzer 2100 High Sensitivity Chip (Agilent; Cat. No. 5067–4626). Each library was then diluted to a concentration of 40 pM and combined with 5 ul of the Ion PGM™ (Personal Genome) Calibration Standard (Cat. No. A27832), as per the protocol for templating libraries with ISPs using the automated Ion Chef (Thermofisher; Chef Package Version IC.5.0.1), and using the Ion PGM Hi-Q Chef Kits (Cat. No. A25948). Finally, we used Ion PGM Hi-Q Chef Kits (Cat. No. A25948) for sequencing with either 318 v2 or 316 v2 chips (Cat. No. 4488146 and 4488145, respectively). Raw data was base-called into fastq formatted files with Torrent Suite Software package v. 5.04, with the calibration standard enabled.

### Transcriptome sequence retrieval, pre-processing and assembly

**Figure 4:**
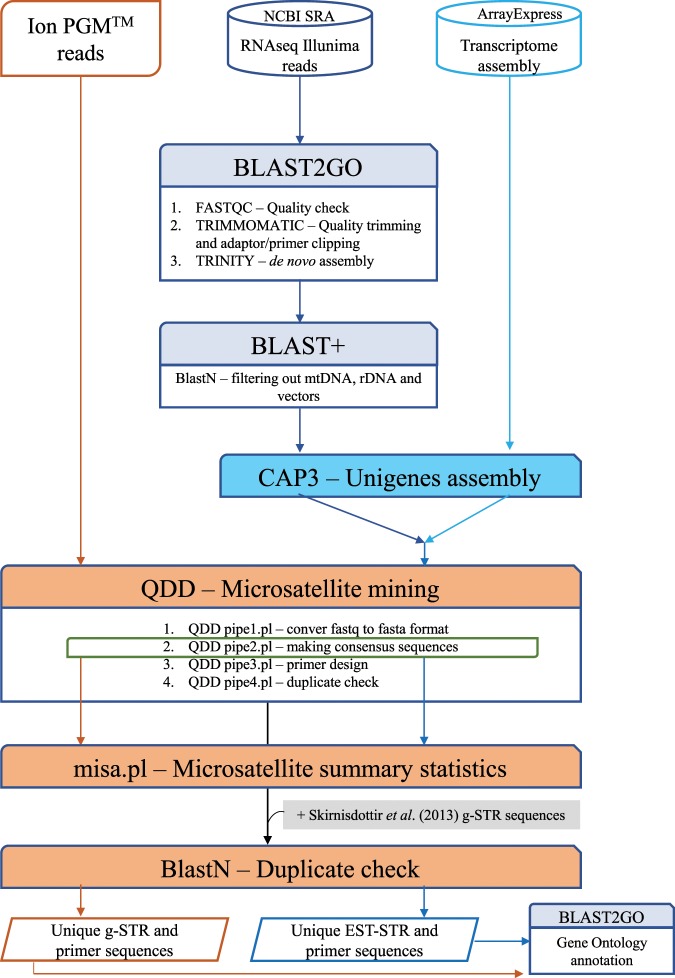
Shematic representation of the bioinformatic analysis used to obtain genome- and transcriptome-derived microsatellite loci for *Cyclopterus lumpus*.

We obtained a recently assembled transcriptome of *C. lumpus* (accession number E-MTAB-6388, Eggestøl *et al*.^47^) from the ArrayExpress Archive of Functional Genomics Data (https://www.ebi.ac.uk/arrayexpress/). In that study, the kidney leukocytes were used to generate RNAseq data for the *de novo* transcriptome assembly. In addition, we downloaded raw RNAseq Illumina paired-end reads of *C. lumpus* (accession number SRX3153215) from the NCBI Sequence Read Archive (SRA) database (https://www.ncbi.nlm.nih.gov/sra) under BioProject PRJNA398732, the Fish-T1K (Transcriptomes of 1000 fishes) Phylogeny Project (Beijing Genome Institute). We submitted the raw sequence reads to a quality control (QC) step in FASTQC as implemented in the BLAST2GO program^64–66^. Next, we conducted a *de novo* transcriptome assembly using the quality-filter sequence data generated from the Fish-T1K project, employing the TRINITY pipeline of Grabherr *et al*.^67^ with the option for read trimming by quality during assembly using TRIMMOMATIC^68^ also implemented in BLAST2GO. Then, we filtered out known contaminants (Vibrio and IPNV), mitochondrial DNA and ribosomal DNA from the assembly using BLAST v. 2.7.1+^69^. Subsequently, we performed further clustering and alignment of each respective transcriptome assembly to form transcript assemblies (unigenes) using the CAP3 program^70^ with parameters *-p* 95, *-o* 49, and *-t* 10 000. Finally, we merged the two transcript assemblies (contigs and singletons) for STR discovery (Fig. 4).

### Microsatellite mining and primer design

To detect and extract STR-containing sequences from the quality-filtered and trimmed ddRADseq dataset, we used the QDD-VM v. 3.2.1 pipeline for low-coverage NGS data^49,71^ (Fig. 4). First, we used the perl script QDD pipe1.pl to convert the input fastq file to fasta and to extract the STR-containing reads with di-to hexanucleotide motifs in both pure (perfect) and compound (imperfect) form, and longer than 80 bp. Second, we used the QDD pipe2.pl script to compare STR-containing reads of each individual using BLAST+, and the reads with very high sequence identity (>95 percent) were grouped into contigs and then used to create a consensus file with CLUSTALW v. 2.0^72^. To construct the consensus sequence for a given locus, we required greater than 0.66 of the sequences to have the same base at a particular site. This step allowed for identifying polymorphic STR loci *i.e*., *in silico* characterisation. Third, we executed the QDD pipe3.pl script to automatically design primers from unique (singletons and consensus) STR-containing sequences using the Primer3 algorithm^73^ implemented within QDD-VM. We used the default parameters for designing primers that Meglécz *et al*.^49^ empirically determined to improve genotyping success rate of STRs and to force the design of primer pairs with variable amplicon size in QDD-VM. The optimised parameters were as follows: product size 90–320 bp; primer size = 18–20–27 bp (min–optimal–max); melting temperature (Tm) = 57–60–63 degrees Celsius (min–optimal–max); GC content = 20–50–80 percent; maximum Tm difference = 10 degrees Celsius. The design of multiple primer pairs with different amplicon size per locus facilitates in silico selection of primer pairs for the design of multiplex PCR during the wet laboratory validation experiments. Last, we executed the QDD *pipe4.pl* script to check for contamination by a BLASTn (query nucleotide against nucleotide database) search (E-value cut-off < 10–20) of all STR-containing sequences with successful primer design against the NCBI database, as well as to compare these sequences to known transposable elements of vertebrates using RepeatMaster v. 4.0.7 (available from http://www.repeatmasker.org/).

To search for EST-STRs we used the QDD-VM pipeline for assembled contigs, which is the same as the above-mentioned pipeline, except for the following parameters: *pipe1.pl* and *pipe3.pl*, -contig was set to 1 to extract STRs with 200 bp flanking regions on both side in the assembled unigenes, *pipe2.pl*, -makecons was set to 0 to avoid paralogs.

Additionally, using the unique STR-containing sequences generated by the QDD *pipe2.pl* script, we executed the perl script *misa.pl* (MIcroSAtellite identifcation tool; available from http://pgrc.ipk-gatersleben.de/misa/) of Thiel *et al*.^74^ to obtain further summary statistics on the identified STRs, which include (i) the distribution of STRs to different repeat unit classes, (ii) the distribution of STRs to different repeat motif length classes, and (iii) the frequency of STR motifs. We defined each repeat motif class of STRs using the MISA specification file (*misa.ini*) with the following parameters: minimum repeat sequence of 10 nucleotides for mononucleotide repeats, and at least five consecutive repeat units for di-, tri-, tetra-, penta- and hexanucleotide motifs. We used the default parameter of less than equal to 100 bp minimum distance between two repetitive units to identify and classify compound repeats. We then estimated the relative abundance of STRs in the transcriptome of *C. lumpus* by dividing the number of STRs found with the total assembly length length (loci/Mb).

Finally, we downloaded the 22 available STR-containing contigs for *C. lumpus* from GenBank (Accession Numbers JX485364–JX485385) and created local BLAST databases for our genomic and transcriptomic datasets, against which we searched for significant hits using BLAST+.

### Functional annotation of contigs containing microsatellites

To classify the putative function of the sequences containing STRs for which primers could be designed, we subjected the EST-derived sequences to a BLASTx (translated query nucleotide against protein database) search with a threshold E-value 10–5 as implemented in BLAST2GO. The three gene ontology (GO) descriptors for functional characterisation of STR-containing sequences were biological process (BP), cellular component (CC), and molecular function (MF). Finally, we executed the mapping function to extract the GO descriptors associated with each of the obtained BLAST hits in BLAST2GO using the annotation cut-off value of 10^−6^. Since our goal was also to provide Type II STRs, we used the same protocol to annotate all g-STR-containing sequences for which primers could be designed, in order to filter out loci associated with coding regions.

## Supplementary information

Supplementary Material
